# Effect of the nitroimidazole Ro 03-8799 on the activity of chemotherapeutic agents against a murine tumour in vivo.

**DOI:** 10.1038/bjc.1984.46

**Published:** 1984-03

**Authors:** P. W. Sheldon, P. Gibson

## Abstract

The effect of the 2-nitroimidazole Ro 03-8799 (8799) on the activity of 11 chemotherapeutic agents against the anaplastic MT tumour in mice has been determined by soft agar cloning. The 8799, whilst producing little cytotoxicity by itself, potentiated the cytotoxic actions of the alkylating agents melphalan and cyclophosphamide, and the nitrosoureas BCNU, CCNU and MeCCNU. This potentiation was influenced by the time interval between the administration of 8799 and the chemotherapeutic agents, and also by the site of tumour implantation. However, 8799 did not potentiate the cytotoxicities of the compounds CBDCA, cisplatin, adriamycin, vincristine, 5-fluorouracil and bleomycin. A review is included of the reported in vivo effects of nitroimidazoles on the chemotherapeutic agents investigated here.


					
Br. J. Cancer (1984), 49, 291-300

Effect of the nitroimidazole Ro 03-8799 on the activity of
chemotherapeutic agents against a murine tumour in vivo

P.W. Sheldon & P. Gibson

Physics Department, Institute of Cancer Research, Clifton Avenue, Sutton, Surrey, SM2 SPX.

Summary The effect of the 2-nitroimidazole Ro 03-8799 (8799) on the activity of 11 chemotherapeutic agents
against the anaplastic MT tumour in mice has been determined by soft agar cloning. The 8799, whilst producing
little cytotoxicity by itself, potentiated the cytotoxic actions of the alkylating agents melphalan and
cyclophosphamide, and the nitrosoureas BCNU, CCNU and MeCCNU. This potentiation was influenced by the
time interval between the administration of 8799 and the chemotherapeutic agents, and also by the site of tumour
implantation. However, 8799 did not potentiate the cytotoxicities of the compounds CBDCA, cisplatin, adriamycin,
vincristine, 5-fluorouracil and bleomycin. A review is included of the reported in vivo effects of nitroimidazoles on
the chemotherapeutic agents investigated here.

Following reports that both in vitro and in vivo,
some chemotherapeutic agents may preferentially
spare hypoxic tumour cells (Hill & Stanley, 1975;
Sutherland et al., 1979), many studies have been
initiated to determine the benefit of combining
these agents with the known hypoxic cell
cytotoxins, nitroimidazoles. In 1979 Kelly et al.
reported that the 5-nitroimidazole, metronidazole,
increased  methotrexate   activity  in   vivo.
Subsequently, many investigators have reported
that the 2-nitroimidazole, misonidazole (miso)
potentiates the activity of many chemotherapeutic
agents against a variety of tumours (see review by
McNally, 1982).

We recently reported potentiation by many
nitroimidazoles of melphalan activity against
intramuscular anaplastic MT tumours in mice
(Sheldon et al., 1982). Maximum potentiation
occurred when the nitroimidazoles were given 0-
30 min before the melphalan. The most effective
compound, Ro 03-8799, was about twice as
effective as miso, and at a dose of 0.72mgg-1 i.p.
enhanced melphalan activity by a factor of 2.2.

We report here on the ability of 8799 to
potentiate another ten anti-cancer drugs. The
importance of the interval between administration
of 8799 and the drugs has been determined. Full
dose response survival curves have been obtained
when the 8799 was given 15 min before the
chemotherapeutic agent and also at the optimum
interval (if any) determined from the time course
studies. As the present work was performed using
s.c. implanted tumours, whereas i.m. implanted
tumours were used in the previous studies, the
influence of tumour site has also been investigated.

Correspondence: P.W. Sheldon, MRC Radiobiology Unit,
Harwell, Didcot, Oxon OXI1 ORD.

Received 13 September 1983; accepted 8 December 1983.

Materials and methods
Mice and tumours

The anaplastic MT tumour was implanted by
injection s.c. over the sacral region of the back of
8-10 week old male inbred WHT/Cbi mice. The
mice were treated when the tumours attained a
mean diameter of 6-7mm (8-11 days after
inoculation).

Cytotoxic agents

All agents were administered i.p. at 0.5ml per 25g
body wt. The agents were always freshly prepared
as follows:

8799 Ro 03-8799, a 2-nitroimidazole supplied by
Dr C.E. Smithen of Roche Products Ltd, was
reconstituted as required in isotonic saline and
administered at a dose of 0.72 mg g- 1.

8800 Ro 03-8800; as for 8799 except that the dose
was 0.73mg g- 1. 8800, used here only to
supplement 8799 data in 2 time course experiments
when further 8799 supply was unavailable, has
previously been shown to be of similar effectiveness
as 8799 at potentiating melphalan (Sheldon et al.,
1982).

CYC Cyclophosphamide 100 by Farmitalia Carlo
Erba Ltd; 100mg vials were reconstituted with 5ml
distilled water injection and diluted as required with
isotonic saline.

BCNU Carmustine by National Institutes of
Health; 100mg vials were reconstituted with 3 ml
ethanol, 27ml distilled water injection added, and
diluted as required with isotonic saline.

CCNU Lomustine by National Institutes of

? The Macmillan Press Ltd., 1984

292   P.W. SHELDON & P. GIBSON

Health; 40 mg capsules were dissolved in 1 ml
DMSO and stored frozen in 0.1 ml aliquots. When
required the aliquots were thawed at room
temperature and diluted as required with 5%
Tween 80 in phosphate buffered saline.

MeCCNU Methyl CCNU by National Institutes
of Health; 50 mg capsules were treated as per
CCNU.

BLEO Bleomycin Lundbeck by Lundbeck Ltd;
15mg vials were diluted as required with isotonic
saline.

cis-Pt Cisplatin by Johnson Matthey Research
Centre, supplied by Dr K.R. Harrap of Department
of Biochemical Pharmacology, Institute of Cancer
Research. The powder was reconstituted as required
in isotonic saline.

CBDCA    Cis-diamine-l,  1-cyclobutane  dicarb-
oxylate platinum (II); obtained and used as for
cisplatin.

5FU 5-Fluorouracil by Roche Products Ltd; Vials
(250 mg) were diluted as required with isotonic
saline.

VCR Vincristine sulphate as Oncovin by Eli Lilly
and Co; 1 mg vials were reconstituted as required
with isotonic saline.

ADR   Adriamycin    doxorubicin   INN     by
Montedison Pharmaceuticals Ltd; 10mg vials were
reconstituted with 5ml distilled water injection and
diluted as required with isotonic saline.

MEL Melphalan as Alkeran by Wellcome
Foundation Ltd; 100mg vials were reconstituted in
5 ml 2% HCI in ethanol and diluted as required in
isotonic saline.

Clonogenic assay

The technique used has been described in detail
previously (Sheldon et al., 1982). Briefly, 2-4
tumour bearing mice were identically treated, 18 h
later  their  tumours  were  excised,  pooled,
enzymically disaggregated into a single cell
suspension, counted, and known numbers of cells
seeded onto soft agar plates. Following 13-15 days
incubation at 37?C the resulting colonies were
counted and their plating efficiency (PE) calculated
from the ratio of the number of colonies counted to
the number of cells seeded.

Results

In the course of the present study, 57 groups of
untreated s.c. tumours and 4 groups of untreated
i.m. tumours were used as controls. Their respective

mean plating efficiencies on incubation were 0.68
(s.d. 0.15) and 0.61 (?0.05), and their respective
mean   cell yields g-1  tumour were   8.6 x 107
(?2.1 x 107) and 1.4x 108 (+4.8 x 107).

Relative to those for the control tumours, the cell
yields g-1 were generally reduced after treatment
with the chemotherapeutic agents (open symbols,
Figure 1). This reduction in cell yield has been
taken into account when expressing tumour
survival. The survival has been expressed as the
surviving   fraction   g1      tumour = relative
P.E. x relative cell yield g- 1.

The survival responses of s.c. tumours to single
doses of the chemotherapeutic agents are shown in
Figure 2. Data for each agent were pooled from 4
or more experiments, and include the drug alone
control responses subsequently obtained during the
8799 studies. The response to each drug did not
appear to differ significantly between experiments.
The survival curves shown in Figure 2 were
computed by least squares fit analysis using the
mathematical relationship S = 1 -(1 -exp (D/Do)"),
with the extrapolation number (n) either not set for
those drugs (i.e. BCNU, CCNU, MeCCNU,
BLEO) whose responses had an initial shoulder
region followed by an exponential decrease in
survival as a function of dose, or set at unity, for
those drugs (i.e. VCR, 5FU, ADR, CisPt, CBDCA,
CYC) whose responses had no shoulder region
before survival decreased exponentially as a
function of dose. It is evident from Figure 2 that
this tumour is responsive to all the ten
chemotherapeutic agents studied.

The effect of 8799 on the response to the
chemotherapeutic  agents   was   initially  in-
vestigated by time course studies. A dose of
chemotherapeutic agent was selected (from Figure
2) that would give about a decade of cell kill, and
the 8799 was administered as a single i.p. dose from
9h before to 8h after the chemotherapeutic agent
(Figures 3 and 4). Though there was much scatter
in the time course data, the optimum interval to
achieve maximum cytotoxicity was taken to be
when the 8799 was administered immediately before
CYC, 30 min before MeCCNU, either 15min or 4 h
before BCNU, 1 h before 5FU, CCNU and
(possibly) VCR. The 8799 appeared to have little
effect on the other agents and hence no optimum
times could be deduced.

The qualitative effects of 8799 described above
were subsequently quantified by derivation of full
dose response curves. The 8799 was administered
either at 15 min or at the optimum time, before the
chemotherapeutic agents. The resulting survival
curves are shown in Figures 5 and 6. The responses
to the chemotherapeutic agents alone have been
redrawn from Figure 2 (omitting data points for
clarity). This was done as the responses to the

EFFECT OF 8799 ON CHEMOTHERAPEUTIC ACTIVITY  293

E

. _

V

0
.)
'a)
a)

1?0                     ADR    o 0                     cis Pt

0                ~~0        .0     0

xi                            xl 0.        ?        ? 00
0                           300                           15

(x "X") Drug dose (mg kg-)

Figure 1 The cell yield g 1 of s.c. tumour obtained after treatment with the chemotherapeutic agents relative
to that for untreated tumours. Saline (0) or 8799 (0) given 15 min before, or 8799 given at "optimum time"
before (U), chemotherapeutic agent. The drug dose for each agent has been scaled as shown by the factor

x.

chemotherapeutic agents did not differ here
between   experiments  and  as  these  curves
represented more data than those available as
concurrent drug alone controls (these data having
been included in Figure 2). The survival curves for
the 8799 plus chemotherapeutic agents in Figures 5
and 6 were computed as described above for the
chemotherapeutic agents alone except that, to allow
for the cytotoxicity of 8799 itself, n was
correspondingly reduced. However, as the 8799
alone toxicity was relatively small (the geometric
mean survival after 8799 alone was 0.73), the
application or otherwise of this correction made no
substantial difference to the conclusions reached.

These were that 8799 did not increase the toxicity
of CBDCA, did give an additive increase with cisPt.
ADR, VCR, BLEO and 5FU, and potentiated the
toxicities of CYC, MeCCNU, CCNU and BCNU.

As this potentiation was relatively small
compared to that reported previously for 8799 in
combination with melphalan (Sheldon et al., 1982),
and as that study employed i.m. tumours, this
combination has been retested here both in s.c. and
i.m. tumours. The responses, shown in Figure 7,
were computed as described above for CYC. The
drug enhancement ratios (DER), calculated from
the ratio of the computed Dos for 8799 plus
melphalan response to melphalan-alone response,

294   P.W. SHELDON & P. GIBSON

E

CD
0
0

.4 _

0)

CD

U,

Drug dose (mg kg-1)

Figure 2 Survival responses of s.c. tumours treated with the chemotherapeutic agents (given 15 min after a
dose of saline).

were 1.5 and 2.0 for s.c. and i.m. tumours
respectively. This difference in DER reflects the
poorer response of i.m. tumours to melphalan alone
since the responses of 8799 in combination with
melphalan are similar for both tumour sites.

Discussion

The effect of the 2-nitroimidazole 8799 on the
activity of chemotherapeutic agents against the
anaplastic MT tumour is summarised in Table I.
Bearing in mind that many drugs do have more
than one mode of action, it can be seen that 8799
did not (according to the sem. of the dose
enhancement ratios) significantly potentiate the

activity of the cross linking CBDCA and cisPt, the
intercalating ADR, the DNA fragmenting BLEO,
the mitotic inhibitor VCR, the anti-metabolite
5FU, but did significantly potentiate MEL, CYC,
BCNU, CCNU and MeCCNU which possess
alkylating activity and, in the case of the
nitrosoureas, also carbamoylating activity.

The failure to observe chemopotentiation by 8799
of the nonalkylating agents is unlikely to be
because of the use of suboptimal intervals since
extensive time course studies were done. Though
these data showed much scatter, there was clearly
no optimum time for the administration of 8799
relative to CBDCA, cisPt, ADR and BLEO, and
although an interval of 60min between 8799 and
5FU and VCR appeared optimal, this is thought to
reflect only their additive toxicities. Of the agents

l

L- 1 (

n

C)

I

EFFECT OF 8799 ON CHEMOTHERAPEUTIC ACTIVITY  295

10-1

3

1-

g 10-1

i

7      _

co

c io-1
.t

CO

:t 10-1
Ui

10-1
lo-2

. I S    CYC

*          C     ---- ?

_4    " .  ,,

4t - - -- -

l~~~~~~~~ q, I

M.CCNU
.  ..0

*:-  @ s   BCNU
0-  .-___
W.  0 ..

*           5FU

;      ---; t@--*   *   .1

0 .4

1o-1

3

0

E io-1

10-  1

C

V,,

I

.2

t; 101

a.-

CD

C

Co 1o-1

.                0.  -

-   _          I :

8       4      0       4       8

Before 4-Time (h) -    After

Figure 3 The effect of a single dose of 8799 given at
various times either before or after a dose of CYC
(25 mgkg- 1),  MeCCNU      (15 mgkg- 1),  BCNU
(30 mg kg- 1),  5FU  (200mgkg-1)    or   CCNU
(10mgkg-1). - -, shows the response to the
chemotherapeutic agent alone derived from Figure 2.

1o-1

I       '     .         cis  Pt

a     U

*  .r
14-0 _ _ _    *    )_ _0  _

* CBDCA

o*g             0
0~~~~~

, 0            0~~

BLEO

.

* 0         _

----  , 0  *

ADR
; -           1- ____

:* :----    0     J%  0

VCR

: _   b    _ t __0

: 0   -  S  *  - - -

S   16o  .

|_.~~

8       4       0      4       8

Befoe - -rime (h) -      After

Figure 4 The effect of a single dose of 8799 (0) or
8800 (M) given at various times either before or after
a dose of cisPt (4mgkg-1), CBDCA (lOOmgkg-9),
BLEO   (500mgkg-1), ADR    (20mgkg-1) or VCR
(2mgkg-1). --, shows the response to the
chemotherapeutic agent alone derived from Figure 2.

CYC

\ \2

120

cisPt

C

5's

"Nk

MeCCNU

N>

e N>

0          $ 60

CCNU

0 \

.\ O

O

100   r     ADR        C   VCR

101            -          0

0            30 0            3

1OUI

0

E

-4.

I

0

.)_

c)
C

*5
.5

co

10 1

lo 2

1001

10

102

0                FO 0              15

Drug dose (mg kg 1)

Figure 5 The effect of 8799 on the survival response

of s.c. tumours to 4 chemotherapeutic agents. Drug
alone responses redrawn from Figure 2 ( ), and
8799 given either 15min (0) or at optimum time (0)
before chemotherapeutic agent.

BCNU

*S

0

I      '&~~,

I        ~~~X,

h        CBDCA

It

K%. 0

N,s

15 0                      15(

BLEO               5FU

0 'N

0

, , 'a 9  *I~~K

0

900 0

450

Drug dose (mg kg 1)

Figure 6 The effect of 8799 on the survival response
of s.c. tumours to 6 chemotherapeutic agents. Drug
alone responses redrawn from Figure 2 ( ), and
8799 given either 15min (0) or at optimum time (0)
before chemotherapeutic agent. (N.B. the optimum
time for BCNU being taken as 4 h.).

10-1

10 -2

L-

0

0 10-3
I

0, 10?

c       -
0)

C5
.5

2 lo-2

lo-3

10 41

-

. . . ._ . .. s

-L

-

I .

s  l    s                s                s                -~~~~~~~~~~~~
. I

* *fI

1                                                               --,6

-i

. *                     |                                         s

-

I   .  -- -- 9

-j

I.                      A

-

I     -          *     - -

I                         _

.1 A 0.

? -0-

I

I                                                              I

. . .

296    P.W. SHELDON & P. GIBSON

100

4 ''sX           SC S

0% 0

10-2      *''8 0        O

0 %

*  % 0

'0   ?

S        0%

10-3             0      0

0p

0@'s      o\

10-4.       .        a         L

0       5        10      151

'    00               IM

*'s           \

0 \

%%    0

0%

0%%

0

*  ',-2. O

0  5   lo   15

0       5       10      15

Melphalan (mg kg-1)

Figure 7 Potentiation by 8799 of melphalan activity against s.c. and i.m. tumours. Saline (0) or 8799 (0)
given 15 min before melphalan. DERs are shown.

that were potentiated, the probable optimum time
to administer 8799 was 4h before BCNU, 60min
before CCNU, 30 min before MeCCNU and
immediately before CYC. For CCNU and
MeCCNU at least, these timings appear critical.
Little, if any, potentiation occurred when the 8799
was given after any of the chemotherapeutic agents.

It can be seen in Table I that (on the basis of
DER + sem) of those chemotherapeutic agents
significantly potentiated against s.c. tumours by
8799, MEL and CCNU were more responsive than
MeCCNU which in turn was more responsive than
either CYC or BCNU. However, the DERs of 1.5
for even the most responsive agents MEL and
CCNU are relatively small compared to those
reported by McNally (1982) in his review of
nitroimidazole potentiation of chemotherapeutic
activity. An explanation for the relatively low
DERs observed in the present studies could be the
routine use of s.c. tumours, for we have also shown
in these studies that for MEL at least, the
potentiation by 8799 is less in s.c. than i.m.
tumours. Another possible explanation, though not
investigated, is that although 8799 has previously
been  reported  to  be   the  most   effective
nitroimidazole examined by us at potentiating
melphalan activity against the MT tumour (Sheldon
et al., 1982), it may not be the most effective for
the other chemotherapeutic agents used here.

The mechanism of nitroimidazole potentiation of
chemotherapeutic  drug  activity  is  currently
receiving much attention. We have previously
shown that for MEL, potentiation of the MT

tumour does not result from nitroimidazole-induced
hypothermia or elimination of the ability to recover
from potentially lethal melphalan damage. Further,
the degree of potentiation is influenced by the
lipophilicity  and  electron-affinity  of  the
nitroimidazoles (Sheldon & Batten, 1982). The
present study shows that the increase in toxocity
when 8799 is combined with chemotherapeutic
agents must occur as a reduction in plating
efficiency at cloning since no reduction in cell yield
at tumour disaggregation is indicated in Figure 1.
This differs from the report by Stephens et al.
(1981) that in the Lewis Lung carcinoma, the
additive  toxicity  that  occurred  when  the
nitroimidazole miso was combined with various
chemotherapeutic agents was expressed as a
reduced cell yield rather than reduced plating
efficiency.

Data from the literature on the in vivo effects of
nitroimidazoles on the chemotherapeutic agents
investigated here are reviewed in Table II. The
objective  of this table is to   show  those
chemotherapeutic   agents   most    frequently
potentiated by nitroimidazoles. For each tumour at
any laboratory only the first reported potentiation
has been cited, irrespective of considerations such
as nitroimidazole, drug dose, or time interval
employed. It can be seen from the lowest row of
the table that there are no reported cases of
potentiation of CBDCA, cisPt or BLEO activities,
potentiation of 5FU, VCR or ADR activities
occurred in a third of the tumours examined, and
potentiation of BCNU, MeCCNU, CCNU, MEL

0

E

0,
C

-W

I

0.
c

0*

CD

(I)

EFFECT OF 8799 ON CHEMOTHERAPEUTIC.ACTIVITY  297

Table I Summary of the effect of 8799 given either 15 minutes or

at the optimum time before the chemotherapeutic agents

D06( ?sem)            DERb( ?sem)

8799              8799
8799      at      8799      at

No.       at      Opt.      at      Opt.
Agent            8799     15min    time     15min    time
CBDCA            50.9      64.5               0.8

(6.6)    (5.8)             (0.1)
cis Pt             1.85     1.83              1.0

(0.03)   (0.05)            (0.0)
ADR               18.6     16.2               1.1

(2.2)    (1.3)             (0.2)
BLEO              309      283                1.1

(36)     (39)              (0.2)

VCR                1.66     1.56     1.45c    1.1      1.1

0.14     0.15     0.24     (0.1)    (0.2)
5FU                1.03    0.93      0.87e    1.1      1.2

(0.08)   (0.04)   (0.10)   (0.1)    (0.2)
CYC               13.0     10.8     9.8c      1.2      1.3

(0.5)    (0.7)    (0.9)    (0.1)    (0.1)
BCNU              9.16      7.61     7.32f    1.2      1.3

(1.20)   (0.33)   (0.53)   (0.2)    (0.2)
CCNU              3.44      2.78     2.25e    1.2      1.5

(0.61)   (0.12)   (0.14)   (0.2)    (0.3)
MeCCNU            6.85      5.02     4.84d    1.4      1.4

(0.48)   (0.20)   (0.21)   (0.1)    (0.1)
MEL (sc)           1.66     1.08     -        1.5      -

(0.14)   (0.11)            (0.2)

MEL (im)          2.34      1.16              2.0      -

(0.19)   (0.12)            (0.3)

aDos?s.e. (mgkg- 1) determined at the computation (as described
above) of the survival curves shown in Figures 5 and 6.

bDERs calculated from Do as described in text.

Optimum times: cimmediately; d30 min; '60 min and f4 h before.

298   P.W. SHELDON & P. GIBSON

- N  0  0  e  0
-O M   o t n - o   cqo
_ Xo N  * N  V-  N N  NI

++ I ++ ++++ ++ I

o N o   6  o  0
-   0  m  e-  N +   "  -  -  n  N

++ +I+++ +I++

n     N

+  * ++  +

++ ++      ++

_ +

+ I

+

+        I +

I I      en r

I  I     I  +

I+       I

N O      N N-     0

I+      +   I     I

N-4N     en         In

I  I     I        I

3to

.   .

+

WI

+4

+ +

VN  N  N
N   N - -

++

+

+q

_

I +

Ifn

~~~~~~~~~-

0

P,

10

o*              I

eI,

"Cl0
r- 0

~ 0
en a

A 00

to-o?

I     o

t. 0o

0 o

- 0
0)

1-0

0 !
0c~

EFFECT OF 8799 ON CHEMOTHERAPEUTIC ACTIVITY  299

or CYC occurred in more than three-quarters of
the tumours examined. Since greater potentiation of
MEL activity has been reported in tumours than
dose-limiting normal tissues (review, McNally,
1982), a therapeutic gain may be anticipated
clinically. It follows from Table II that the most
promising    chemotherapeutic   agents    for
consideration for clinical trial in combination with
nitroimidazoles are the alkylating agents, be they
the direct acting MEL or enzymically activated
CYC (Sladek, 1973), or the nitrosoureas BCNU,
MeCCNU or CCNU.

We wish to thank Drs K.K. Fu, T.L. Phillips, P.A. Raynor
and G.Y. Ross for permission to cite their unpublished
data; Miss D.J. Scottow and Mr T.J. Madigan for
excellent animal husbandry: Miss E.L. Batten for technical
assistance; Prof G.E. Adams and Drs K.R. Harrap and
T.C. Stephens for advice in the preparation of this
manuscript; and the MRC and NCI (contract grant no.
NOI-CM-17502) for financial support.

References

CLEMENT, J.J., GORMAN, M.S., WODINSKY, I., CATANE,

R. & JOHNSON, R.K. (1980). Enhancement of
antitumour activity of alkylating agents by the
radiation sensitiser misonidazole. Cancer Res., 40,
4165.

CLEMENT, J.J. & JOHNSON, R.K. (1982). Evaluation of

radiosensitisers in combination with chemotherapeutic
agents in solid tumours. Int. J. Radiat. Oncol. Biol.
Phys., 8, 631.

CLUTTERBUCK, R.D., MILLAR, J.L. & McELWAIN, T.J.

(1982). Misonidazole enhancement of the action of
BCNU and melphalan against human melanoma
xenografts. Am. J. Clin. Oncol., 5, 73.

FU, K.K., PHILLIPS, T.L., RAYNER, P.A. & ROSS, G.Y.

(1982). Combined effects of misonidazole and
chemotherapeutic drugs on tumour and normal tissue
in vivo. (Personal communication.)

HILL, R.P. & STANLEY, J.A. (1975). The response of

hypoxic B16 melanoma cells in vitro treatment with
chemotherapeutic agents. Cancer Res., 35, 1147.

HIRST, D.G. & BROWN, J.M. (1982). The therapeutic

potential of misonidazole enhancement of alkylating
agent cytotoxicity. Int. J. Radiat. Oncol. Biol. Phys., 8,
639.

HIRST, D.G., BROWN, J.M. & HAZLEHURST, J.L. (1982).

Enhancement of CCNU cytotoxicity by misonidazole:
possible therapeutic gain. Br. J. Cancer, 46, 109.

KELLY, J.P., HANNAM, T.W. & GILES, G.R. (1979). The

cytocidal action of metronidazole in combination with
other antineoplastic agents. Cancer Treat. Rev., 6
(Suppl.), 53.

LAW, M.P., HIRST, D.G. & BROWN, J.M. (1981). The

enhancing effect of misonidazole on the response of
the RIF-I tumour to cyclophosphamide. Br. J. Cancer,
44, 208.

MARTIN, W.M.C., McNALLY, N.J. & DE RONDE, J. (1981).

Enhancement of the effect of cytotoxic drugs by
radiosensitizers. Br. J. Cancer, 43, 756.

McNALLY, N.J. (1982). Enhancement of chemotherapy

agents. Int. J. Radiat. Oncol. Biol. Phys., 8, 593.

McNALLY, N.J., HINCLIFFE, M. & DE RONDE, J. (1983).

Enhancement of the action of alkylating agents by
single high or chronic low doses of misonidazole. Br.
J. Cancer, 48, 271.

MULCAHY, R.T. (1982). Chemical properties of nitroureas:

implications for interaction with misonidazole. Int. J.
Radiat. Oncol. Biol. Phys., 8, 599.

MULCAHY, R.T., SIEMANN, D.W. & SUTHERLAND, R.M.

(1981). In vivo response of KHT sarcomas to
combination chemotherapy with radiosensitizers and
BCNU. Br. J. Cancer, 43, 93.

MURRAY, D. & MEYN, R.E. (1983). Enhancement of the

DNA cross-linking activity of melphalan by
misonidazole in vivo. Br. J. Cancer, 47, 195.

PEDERSEN, J.E., BARRON, G. & MEEKER, B.E. (1982).

The   value  of   combining  the   radiosensitiser
misonidazole with cyclophosphamide in treating the
murine Lewis Lung tumor. Int. J. Radiat. Oncol. Biol.
Phys., 8, 651.

RANDHAWA, V.S., STEWART, F.A. & DENEKAMP, J.

(1982). Chemosensitisation of mouse tumours by
misonidazole. Int. J. Radiat. Oncol. Biol. Phys., 8, 671.

ROSE, C.M., MILLAR, J.L., PEACOCK, J.H., PHELPS, T.A. &

STEPHENS, T.C. (1980). Differential enhancement of
melphalan cytotoxicity in tumour and normal tissue by
misonidazole. In Radiation Sensitizers, p. 250. (Ed.
Brady). New York: Masson.

SHELDON, P.W. & BATTEN, E.L. (1982). Potentiation in

vivo  of  melphalan  activity  by  nitroimidazole
compounds. Int. J. Radiat. Oncol. Biol. Phys., 8, 635.

SHELDON, P.W., BATTEN, E.L. & ADAMS, G.E. (1982).

Potentiation of melphalan activity against a murine
tumour by nitroimidazole compounds. Br. J. Cancer,
46, 525.

SIEMANN, D.W. (1981). In vivo combination of

misonidazole and the chemotherapeutic agent CCNU.
Br. J. Cancer, 43, 367.

SIEMANN, D.W. & MULCAHY, R.T. (1982). Cell survival

recovery kinetics in the KHT sarcoma following
treatment with five alkylating agents and misonidazole.
Int. J. Radiat. Oncol. Biol. Phys., 8, 619.

SLADEK, N.E. (1973). Bioassay and relative cytotoxic

potency of cyclophosphamide metabolites generated in
vitro and in vivo. Cancer Res., 33, 1150.

STEPHENS, T.C., COURTENAY, V.D., MILLS, J., PEACOCK,

J.H., ROSE, C.M. & SPOONER, D. (1981). Enhanced cell
killing in Lewis Lung carcinoma and a human
pancreatic-carcinoma xenograft by the combination of
cytotoxic drugs and misonidazole. Br. J. Cancer, 43,
451.

SUTHERLAND, R.M., EDDY, H.A., BAREHAM, B., REICH,

K. & VANANTWERP, D. (1979). Resistance to
adriamycin in multicellular spheroids. Int. J. Radiat.
Oncol. Biol. Phys., 5, 1225.

300   P.W. SHELDON & P. GIBSON

TANNOCK, I.F. (1980a). In vivo interaction of anti-cancer

drugs   with   misonidazole  or    metronidazole:
methotrexate, 5-fluorouracil and adriamycin. Br. J.
Cancer, 42, 861.

TANNOCK, I.F. (1980b). In vivo interaction of anti-cancer

drugs   with   misonidazole  or    metronidazole:
cyclophosphamide and BCNU. Br. J. Cancer, 42, 871.

TWENTYMAN, P.R., (1981). Modification of tumour and

host response to cyclophosphamide by misonidazole
and by WR 2721. Br. J. Cancer, 43, 745.

TWENTYMAN, P.R. & WORKMAN, P. (1982). The effects

of radiosensitiser pretreatment on the response of the
RIF-1 mouse sarcoma to cytotoxic drugs. Int. J.
Radiat. Oncol. Biol. Phys., 8, 611.

TWENTYMAN, P.R. & WORKMAN, P. (1983).

Chemosensitisation by lipophilic nitroimidazoles. Br.
J. Cancer, 48, 17.

WODINSKY, I., JOHNSON, R.K. & CLEMENT, J.J. (1979).

Enhanced activity against murine tumours of
cyclophosphamide in combination with the hypoxic
cell radiosensitiser misonidazole. Proc. Am. Assoc.
Cancer Res., 20, 230.

WORKMAN, P. & TWENTYMAN, P.R. (1982).

Enhancement by electron-affinic agents of the
therapeutic effects of cytotoxic agents against the KHT
tumour: structure-activity relationships. Int. J. Radiat.
Oncol. Biol. Phys., 8, 623.

				


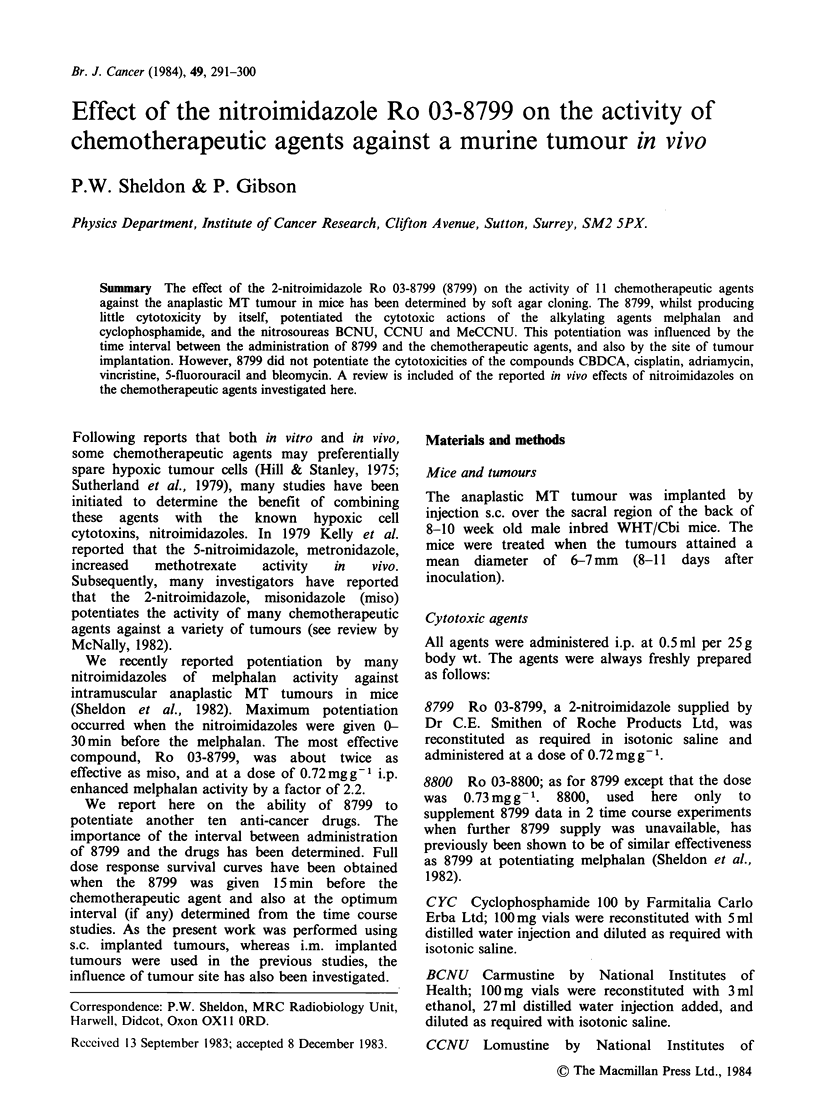

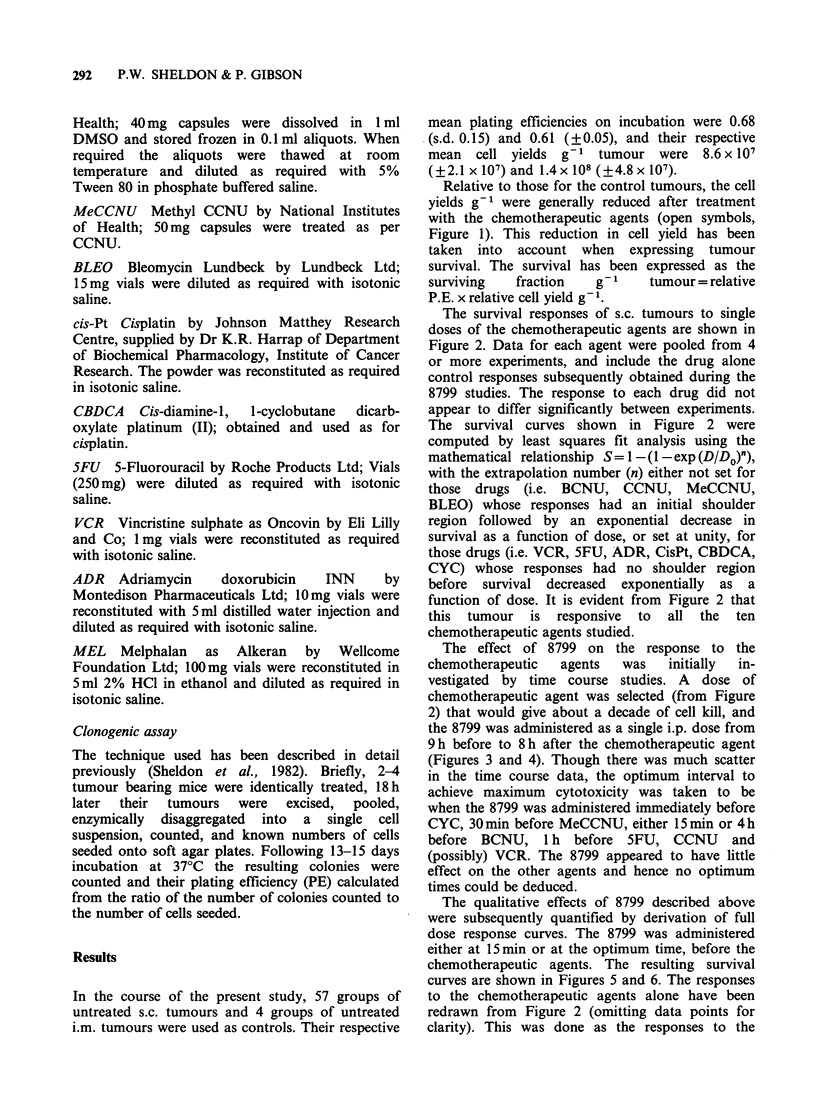

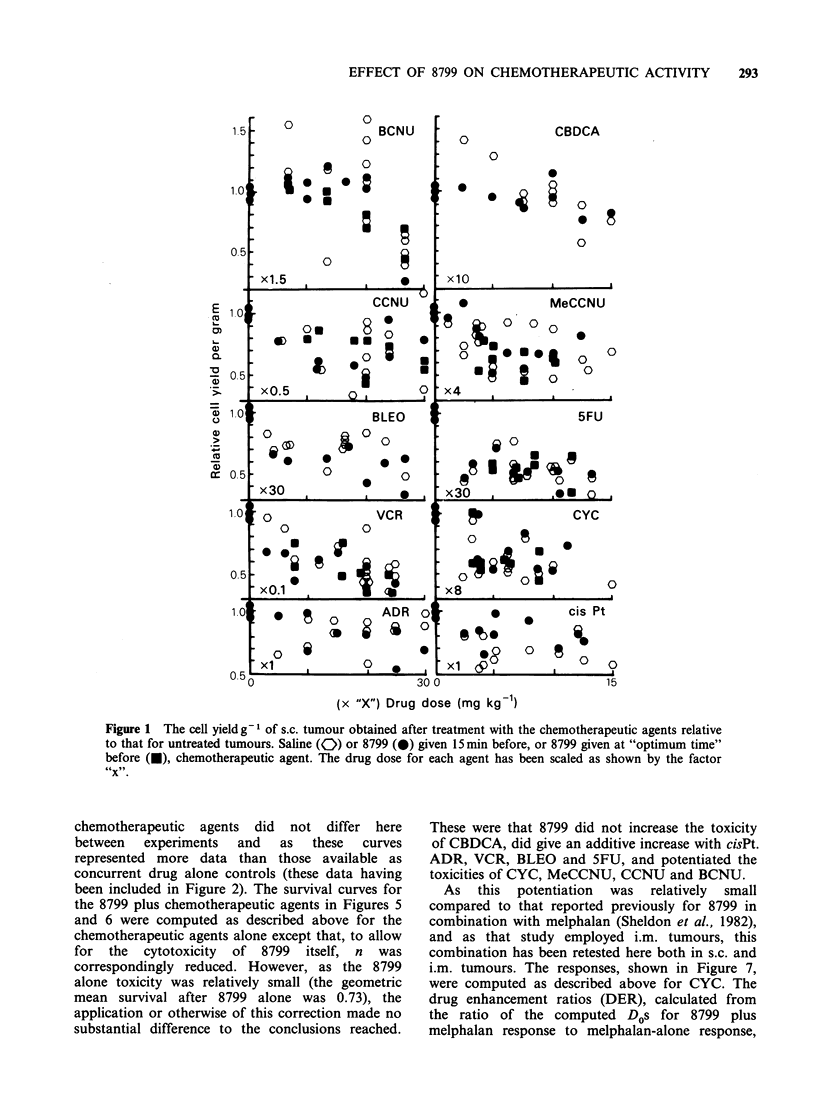

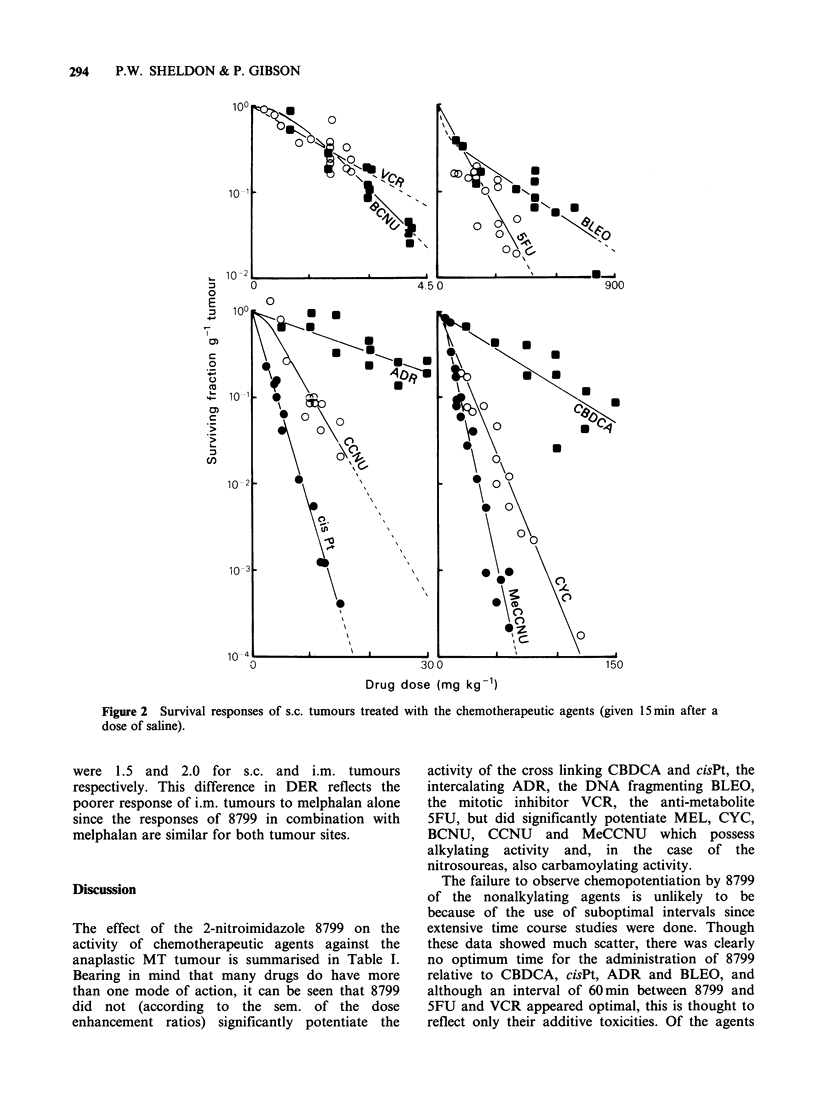

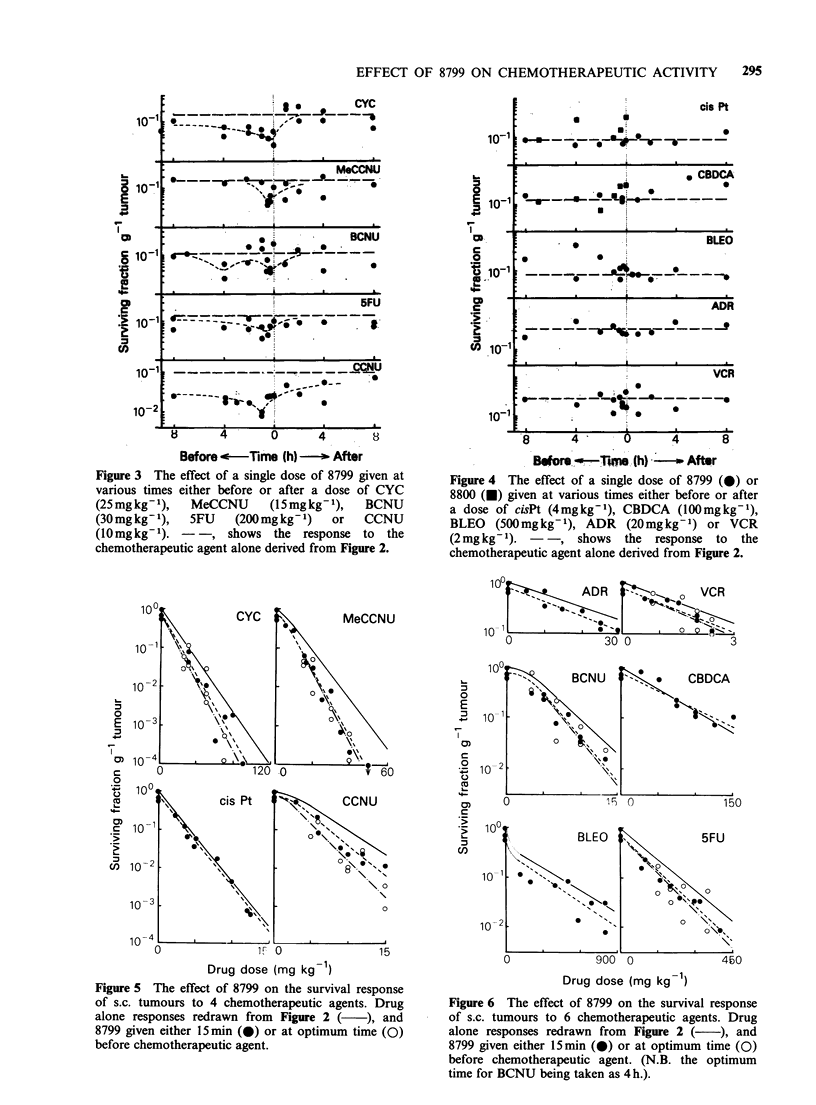

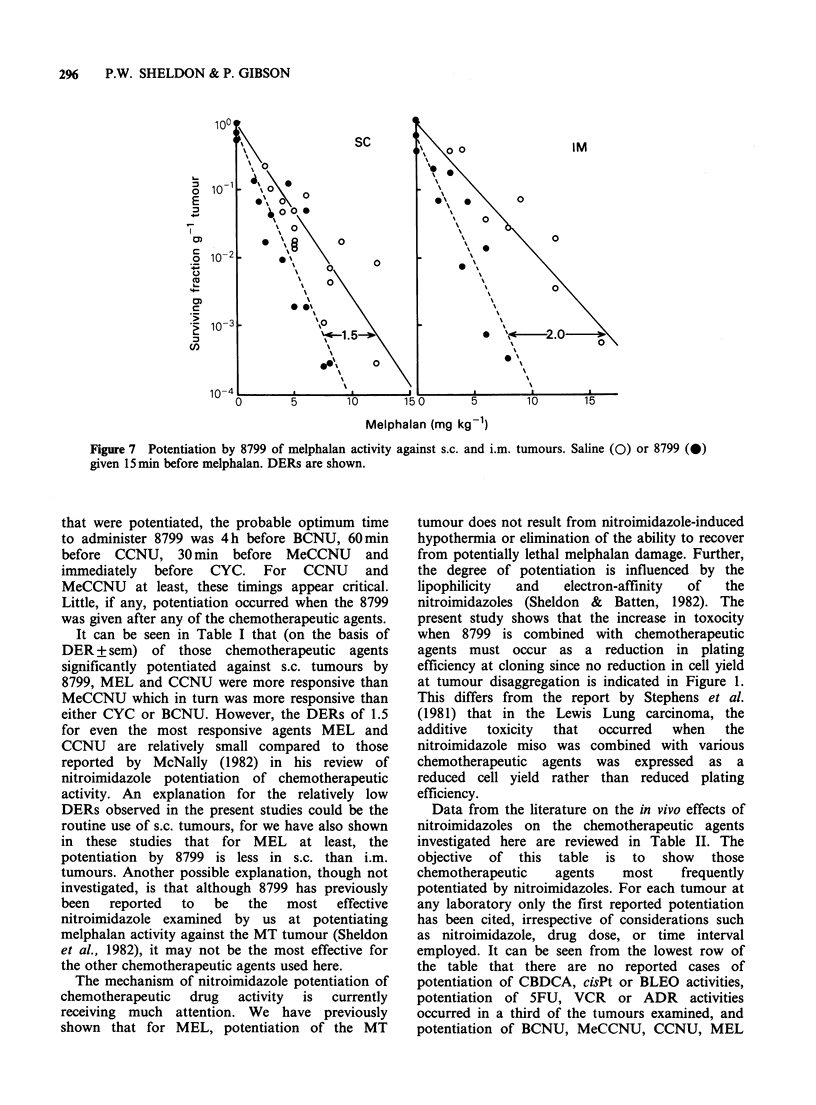

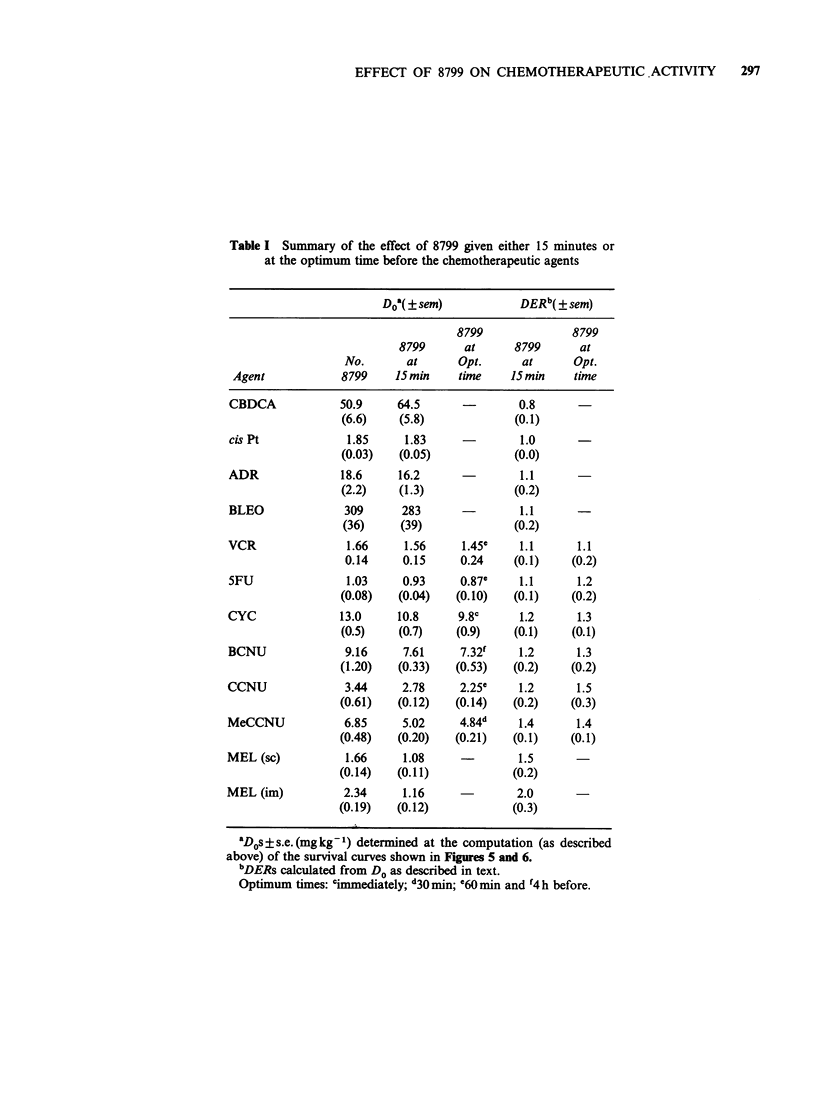

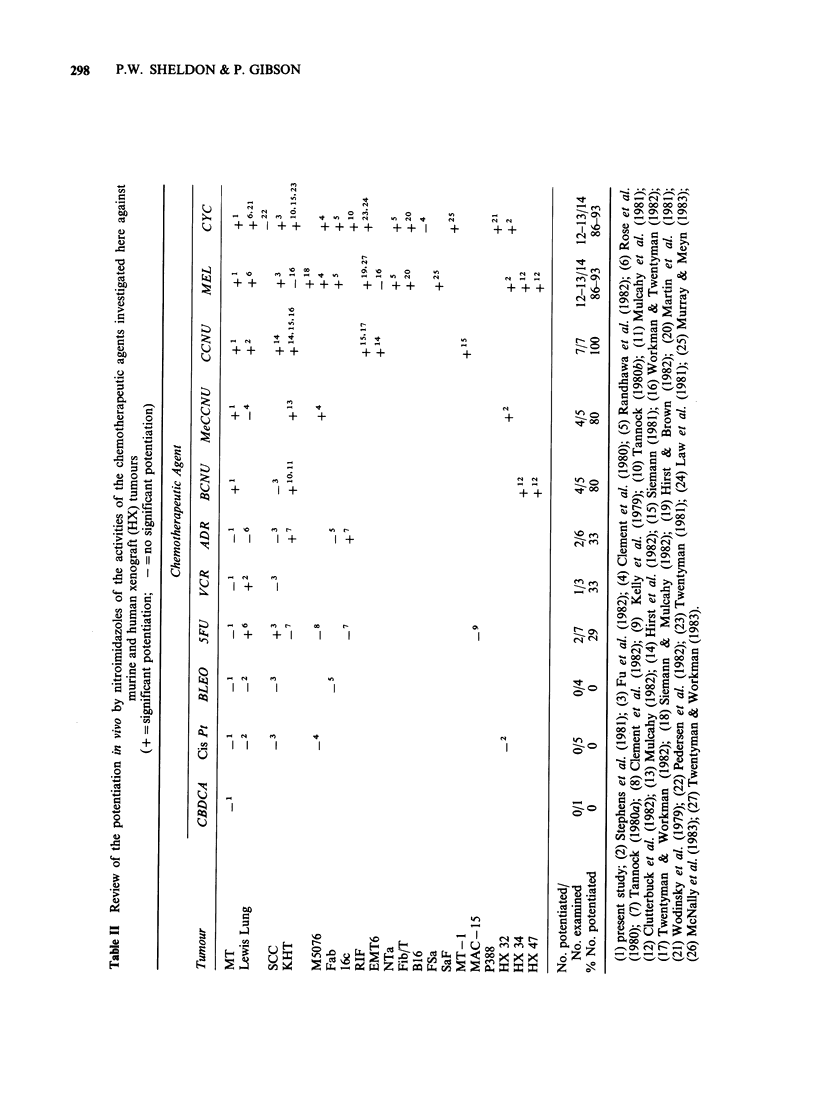

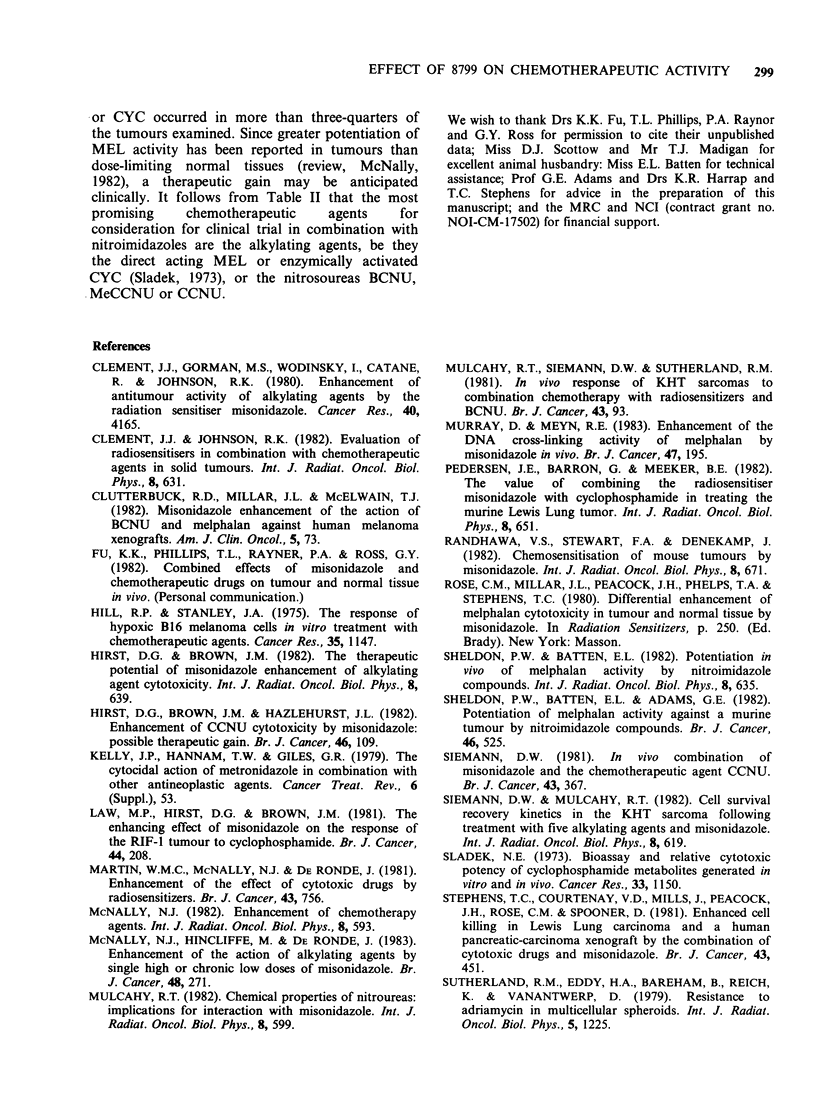

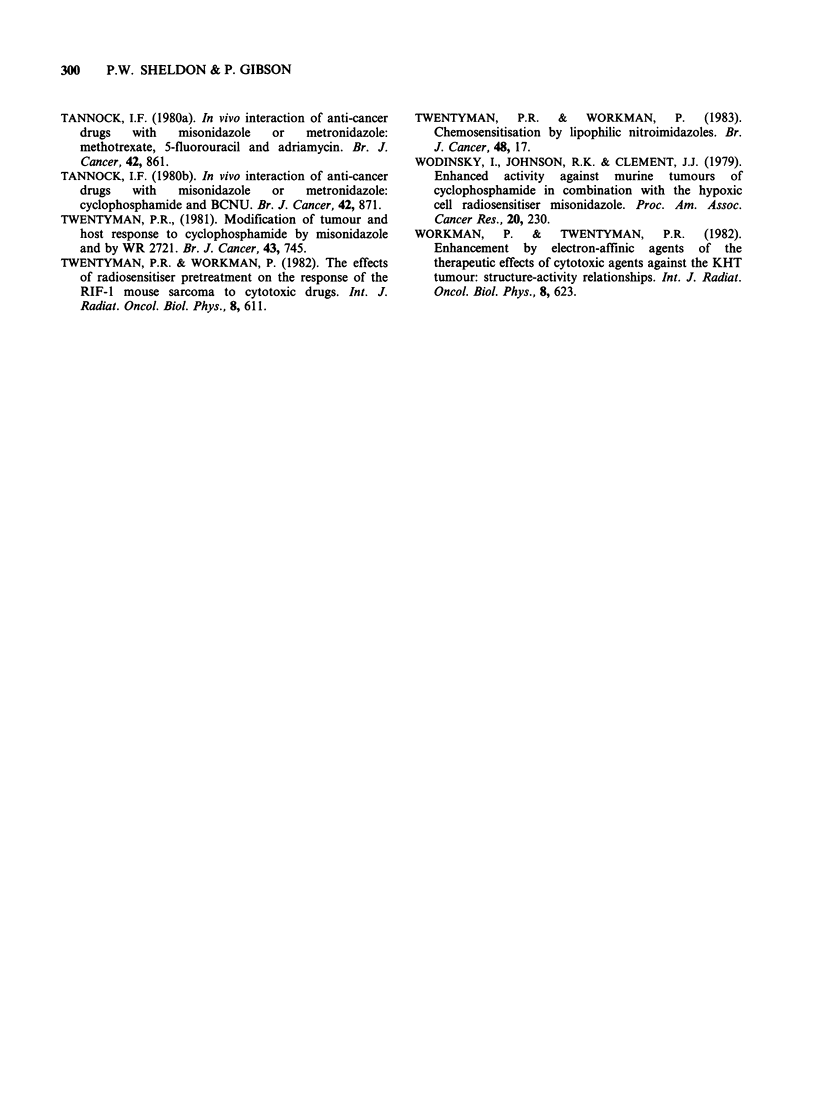

